# The Built Environment Predicts Observed Physical Activity

**DOI:** 10.3389/fpubh.2014.00052

**Published:** 2014-05-20

**Authors:** Cheryl Kelly, Jeffrey S. Wilson, Mario Schootman, Morgan Clennin, Elizabeth A. Baker, Douglas K. Miller

**Affiliations:** ^1^Department of Health Sciences, University of Colorado Colorado Springs, Colorado Springs, CO, USA; ^2^Department of Geography, School of Liberal Arts, Indiana University-Purdue University Indianapolis, Indianapolis, IN, USA; ^3^College for Public Health and Social Justice, Saint Louis University, St. Louis, MO, USA; ^4^Department of Exercise Science, Arnold School of Public Health, University of South Carolina, Columbia, SC, USA; ^5^Regenstrief Institute Inc., Center for Aging Research, Indiana University School of Medicine, Indianapolis, IN, USA

**Keywords:** walkable, micro characteristics, street view, objective measures, policy interventions

## Abstract

**Background:** In order to improve our understanding of the relationship between the built environment and physical activity, it is important to identify associations between specific geographic characteristics and physical activity behaviors.

**Purpose:** Examine relationships between observed physical activity behavior and measures of the built environment collected on 291 street segments in Indianapolis and St. Louis.

**Methods:** Street segments were selected using a stratified geographic sampling design to ensure representation of neighborhoods with different land use and socioeconomic characteristics. Characteristics of the built environment on-street segments were audited using two methods: in-person field audits and audits based on interpretation of Google Street View imagery with each method blinded to results from the other. Segments were dichotomized as having a particular characteristic (e.g., sidewalk present or not) based on the two auditing methods separately. Counts of individuals engaged in different forms of physical activity on each segment were assessed using direct observation. Non-parametric statistics were used to compare counts of physically active individuals on each segment with built environment characteristic.

**Results:** Counts of individuals engaged in physical activity were significantly higher on segments with mixed land use or all non-residential land use, and on segments with pedestrian infrastructure (e.g., crosswalks and sidewalks) and public transit.

**Conclusion:** Several micro-level built environment characteristics were associated with physical activity. These data provide support for theories that suggest changing the built environment and related policies may encourage more physical activity.

## Introduction

Because of physical activity’s relationship to health, researchers have been evaluating the association between physical activity and the built environment. Despite increasing evidence suggesting the built environment is associated with increased physical activity, research to date is inconclusive on the exact role of the built environment as it relates to physical activity and which specific aspects of the built environment are most influential (1–3).

One of the current limitations in the field is the inability to directly compare results across different studies due to inconsistencies in the methods or technologies used to measure both the built environment and physical activity (2, 4). For example, using different approaches to measure physical activity (i.e., self-reported or observed) greatly influenced the consistency of associations between environmental attributes and youth physical activity, with observed built environment measures and self-report physical activity measures demonstrating the most consistent associations (2). However, self-report physical activity may introduce recall bias and social desirability issues, potentially leading to over or under estimation of actual behavior (5, 6).

Another limitation is that studies often do not directly link physical activity with specific geographic location (3, 7). A common method is to use surveys that ask respondents to recall their activity for the last week or month. Researchers then either assume that the activity occurred in or near the respondents’ homes or work, ask respondents to recall the location (e.g., home or a gym) of that activity, or assume that the activity happened within a certain distance (or buffer) around that location (e.g., 400 m). These methods introduce bias and uncertainty. For example, asking respondents to identify locations of physical activity introduces recall and social desirability biases (8). As Ding and Gebel suggest, studies that do not match the purpose of physical activity with specific environmental attributes where the activity was actually performed may miss important associations (i.e., type-2 errors) (3).

To understand which built environment characteristics are the most significant predictors of physical activity behavior, new methods and emerging technologies allow researchers to assess behavior unobtrusively to circumvent self-report biases and directly link that behavior with attributes of specific geographic locations. Recently, researchers have developed direct observation methods that systematically capture behavior as it is occurring (9–13). For example, the Block Walk Method, an observational method used to identify physical activity as it occurs on streets and sidewalks, provides evidence as to whether the physical activities of interest were performed in the environments being examined (9, 11, 12). Observational methods have several advantages over survey methods. First, they remove respondent bias by unobtrusively monitoring physical activity behavior. Second, they allow researchers to identify the type of physical activity, as well as when, where, and with whom it occurs (13). Recent studies have demonstrated improved estimation of the effects of built environment on physical activity when this type of specificity is incorporated (14). Additionally, using observational methods to evaluate the built environment and for physical activity assessments prevents any opportunity for same-source bias, which could bias the association away from the null (15, 16).

Assessing physical activity behavior unobtrusively directly in the geographic context in which it occurs allows researchers to better understand which characteristics of the built environment predict behavior. Emerging technology using high-resolution omnidirectional imagery is a reliable and efficient method to assess the built environment (17–22). Omnidirectional imagery refers to the simultaneous collection of images in multiple directions from a single location, producing a 360° panoramic view. This imagery provides a permanent visual record of an area and allows the viewer to virtually observe characteristics that are included on many built environment audit instruments. Google Street View^1^ and Microsoft Virtual Earth^2^ are probably the most well-known examples of publicly accessible omnidirectional imagery. Many built environment characteristics that were previously measured only through direct observation are now revealed in publicly accessible imagery. Recent research by the authors found high agreement between built environment audits conducted with imagery sources (including Street View) and field audits (mean agreement of 0.81), indicating imagery is a reliable and potentially more efficient alternative to field audits for many built environment features (18). Additionally, the authors recently reported substantial to nearly perfect inter-rater reliability when using Street View imagery to audit built environment items included on the Active Neighborhood Checklist (17). Linking these new methods (i.e., built environment audits using publicly accessible imagery and direct observation of behavior) is potentially important for advancing our understanding of the relationships between the built environment, physical activity, and related health outcomes.

### Purpose

The purpose of this study was to assess the relationship between built environment characteristics using field audits (the gold standard) and imagery audits (emerging technology) and physical activity using a direct observation method, directly linking behavior with the specific geographic location and attributes of the built environment.

## Materials and Methods

### Sampling

Four hundred street segments in suburban and urban areas in Indianapolis, Indiana and St. Louis, Missouri were sampled for inclusion in this study. A street segment was defined as the section of the road between two consecutive intersections. Two hundred segments in each city were selected using a stratified random geographic sampling design to ensure representation of neighborhoods with different land use and socioeconomic characteristics. The percent of the total area in commercial land use on each street segment was estimated using parcel-level land use data provided by local government agencies. Segments were classified as above or below the median percent area of commercial land use; medians were calculated separately in each city. A previously established method for socioeconomic stratification using two race categories (>50% African American or >50% White) and two income categories based on the percentage of population in poverty (low poverty <10.0% and high poverty ≥20.0%) was also applied to each segment (18). This sampling method resulted in eight strata; 25 segments in each city were selected randomly within each stratum.

### Data collection

Characteristics of the built environment were assessed using two objective methods: field audits and imagery audits. While field audits have been the gold standard for assessing the built environment, new methods using high-resolution omnidirectional imagery to assess the built environment have recently been established (17–22).

Field and imagery audits of built environment characteristics were conducted using the Active Neighborhood Checklist (23). The Checklist includes 89 items across six domains assessing presence or absence of land use characteristics, public transportation, street characteristics, quality of the environment for pedestrians, sidewalks and related features, shoulders, and bike lanes.

Multiple teams of two research assistants in each city were trained to conduct built environment audits. Prior to conducting audits, research assistants participated in a 4-h training that included conducting practice audits on segments with varying built environment characteristics (23). Auditors then reviewed their results with each other to discuss any discrepancies. The same process was used to practice built environment audits using Google Street View imagery. Training materials used for the field and imagery audit training are available online at www.activelivingresearch.org/node/10616. The St. Louis team conducted the field audits for street segments in St. Louis, and the imagery audits for Indianapolis blinded to results obtained by the Indianapolis team (and vice-versa for the Indianapolis team) to avoid same-source bias (16).

Two teams of two observers in each city (four teams total) participated in a 5-h training to assess physical activity behavior via direct observation. Observers were trained to use the Block Walk Method, a reliable recording tool and training method (9, 11, 12). This method entails having observers walk both sides of the street at 30.5 m/min pace (with the aid of a metronome), while identifying physical activity behavior that occurs in the observation field (on either side of the street). The observation field was defined as extending to the left and right of the observer’s shoulders (9). Physical activity was recorded if the observer crossed a parallel plane of motion to an observed physical activity [i.e., the observer crossed paths with the physically active person(s)]. For more information on the Block Walk Method, see Suminski et al. (9). Physical activity was captured using a multiple-tally denominator click counter with individual counters for each category of physical activity (walking, biking, running, walking a dog, and other). Different teams of two conducted the built environment audits and physical activity behavior observations (i.e., the built environment auditors did not conduct direct observation of physical activity behavior on the same segments).

Direct observation of behavior was conducted on each segment four times on varying days of the week and times of the day. Segments were allotted to each team by first clustering segments based upon proximity to each other (with one to five segments per cluster). The clusters were then divided between the two teams. Each team’s clusters were randomly assigned 2 weekday and 2 weekend observations and randomly allocated between hours in the morning to afternoon (between 9:00 a.m. and 3:00 p.m.) and in the early evening (between 4:00 and 7:00 p.m.) using a random number generator. The goal of this random assignment was to spread the assessments across different days and times of day. Teams typically observed three clusters for every 4-h shift, with a half hour allotted for travel time to and from different street segments.

Of the 400 initially sampled segments, 24 segments in Indianapolis and 103 in St. Louis were not covered by Google Street View imagery at the time the study was conducted. To maintain enough statistical power, 62 additional segments were selected in St. Louis using the same sampling criteria. Another 45 segments in both cities were not audited in the field due to safety concerns of the auditors, problems with identifying the specific segment in the field, or scheduling issues with auditors. The final analytical sample included 291 segments (153 in Indianapolis and 138 in St. Louis) with all three sources of data (i.e., field audits, imagery audits, and direct observation of behavior). Table 1 summarizes the initial streets sampled and the final analytic sample of 291 segments.

**Table 1:** **Final analytic sample of segments by race, income, and land use stratification (*n* = 291)**.

	St. Louis	Indianapolis	Total sampled	Streets excluded
1. >50% African American, high poverty, above median commercial land use	15 (11%)	17 (11%)	32	20
2. >50% African American, high poverty, below median commercial land use	18 (13%)	20 (13%)	38	22
3. >50% African American, low poverty, above median commercial land use	18 (13%)	18 (12%)	36	25
4. >50% African American, low poverty, below median commercial land use	17 (14%)	23 (15%)	40	22
5. >50% White, high poverty, above median commercial land use	16 (12%)	18 (12%)	34	22
6. >50% White, high poverty, below median commercial land use	16 (12%)	18 (12%)	34	30
7. >50% White, low poverty, above median commercial land use	20 (14%)	19 (13%)	39	3
8. >50% White, low poverty, below median commercial land use	19 (14%)	19 (13%)	38	3
	139	152	291	147

### Statistical analyses

Consistent with previous studies using the Checklist, items that required auditors to indicate if something was present on one side of the segment, both sides of the segment, or not present were characterized as present (on one or both sides) versus absent (17, 18, 23). Additionally, ordinal items (i.e., none, some, or a lot for litter and tree shading; and flat, moderate, or steep for slope) were dichotomized as present (some/a lot or moderate/steep) versus absent (none or flat). A total of 58 Checklist items had adequate data and were included in the analysis. Segments were categorized as having a particular characteristic (e.g., sidewalk present or not) based on field audit results and again based on image-based results. The following Checklist items were excluded from analyses because of lack of observed variability across the audited segments: presence of large apartment buildings (>4 stories), apartment over retail, basketball/tennis/volleyball court, playground, outdoor pool, entertainment, library or post office, laundry facility, indoor fitness center, college or university, high rise building, big box store or mall, supermarket, bench/shelter at transit stop, sidewalk through a cul-de-sac, public art, off-road trail, alternative places to walk or bike, and sidewalk and shoulder permanent obstructions (≤5 streets with this characteristic). Alternative places to walk and shoulder obstructions were only assessed if there was not a sidewalk, contributing to the lack of variability.

Counts of individuals engaged in different types of physical activity behavior observed on each segment were summed across the four observation periods and by behavior type. This resulted in separate counts of the number of walkers, bikers, runners, individuals with a dog (running or walking), and other (e.g., skateboard and roller blades) for each segment. A total behavior variable was calculated by summing across all types of physical activity behavior categories.

Because the counts of physically active individuals were not distributed normally, non-parametric statistics were used. Mann–Whitney *U* tests were used to compare median number of walkers, bikers, runners, people with a dog, and total behavior on segments with each built environment characteristic present to segments without such characteristics present (dichotomous variable). Kruskal–Wallis tests were used to compare physical activity behavior on type of land use: all residential, mixed land use, or non-residential land use (categorical variable with three response choices). Additionally, to assess if the results varied by population density, the Kruskal–Wallis test was used to assess if there was a significant difference in built environment characteristics by persons per square mile (US Census data). All analyses were completed separately for field audits and image-based audits.

## Results

The number of observed walkers, bikers, runners, individuals with a dog, and other activity are summarized in Table 2. Because the prevalence of bikers, runners, and individuals with a dog, and in other activities on each segment was low (median = 0), they were not analyzed separately. However, they were included in the total physical activity behavior category. The remainder of this paper focuses on the total number of physically active persons, regardless of type of activity.

**Table 2:** **Number of individuals observed being physically active on 291 segments**.

	Mean (SD)	Median	Range
Walkers	3.7 (14.8)	1.0	0–210
Bikers	1.0 (1.9)	0.0	0–14
Runners	0.2 (0.9)	0.0	0–11
With a dog	0.2 (0.6)	0.0	0–4
Other	0.1 (0.4)	0.0	0–4
Total physical activity	5.3 (15.7)	2.0	0–219

Table 3 shows the relationship between 58 of the built environment characteristics derived from field audits and imagery audits with total physical activity (sum individuals engaged in all types of physically active behavior). There were significantly more physically active individuals observed on segments with certain built environment characteristics. However, because agreement between field and image-based audits was not 100% on all items, some streets were classified as having a built environment characteristic using field audits, while the same street was classified as not having that characteristic when using image-based audits. This disagreement affected the results for 28% (*n* = 16) of the items. For example, when assessing the presence of abandoned homes, medium or large parking lot or garage, abandoned buildings, food establishments, posted special speed zone, amenities, slope along walking area, sidewalk width of at least 5 ft, major sidewalk misalignments, and shoulder width at least 4 ft, there was significantly more physical activity on these streets when assessed by field audits but not when assessed by imagery audits. Similarly, when assessing the presence of undeveloped land, apartments (>4 units), banks, a median/island, and a turn lane, there was significantly more physical activity on these streets when assessed by imagery audits but not when assessed by field audits. However, despite disagreement in significance, the median number of physically active individuals was in the same direction for all of these items (i.e., regardless of auditing method, more physically activity individuals were observed on segments with certain characteristics). However, for one item, sidewalk width at least 3 ft, there was significantly more physical activity observed on streets without this characteristic when assessed by field audits (median = 3.0) but significantly more physical activity observed on streets with this characteristic when assessed by imagery audits (median = 5.0).

**Table 3:** **Total individuals engaged in physically active behavior on streets with specific built environment characteristics^a,b^**.

		Streets assessed with field			Streets assessed with imagery		
		No. of streets	Mean (SD)	Median	No. of streets	Mean (SD)	Median
**LAND USE**							
All residential		172	2.8 (4.5)	1.0	173	3.0 (4.7)	1.0
Mixed land use		95	7.5 (22.5)	4.0	88	7.3 (23.4)	4.0
All non-residential		24	13.8 (27.1)	5.0**	30	12.2 (24.5)	5.0**
**PREDOMINANT LAND USE PRESENT**							
Residential building	No	28	13.1 (25.3)	5.0*	36	16.9 (41.3)	5.0**
	Yes	263	3.0 (13.2)	1.0	255	3.6 (4.9)	2.0
Commercial building	No	219	3.5 (4.9)	2.0	**218**	**3.4 (4.8)**	**2.0**
	Yes	72	10.6 (29.9)	4.0*	**73**	**10.7 (29.7)**	4.0**
School/school yards	No	**276**	**5.0 (15.9)**	**2.0**	**276**	**5.1 (16.0)**	**2.0**
	Yes	**15**	**9.5 (8.8)**	6.0**	**15**	**7.8 (7.2)**	7.0**
Parking lots or garages	No	276	5.2 (16.0)	2.0	267	5.0 (16.1)	2.0
	Yes	15	6.7 (8.5)	4.0	24	7.9 (9.9)	3.5
Park with equipment	No	282	4.5 (9.5)	2.0	286	5.2 (15.8)	2.0
	Yes	9	28.2 (67.2)	7.0*	5	8.4 (5.0)	8.0*
Vacant lots/abandoned buildings	No	270	5.1 (16.2)	2.0	271	5.3 (16.2)	2.0
	Yes	11	6.9 (7.2)	6.0	20	5.3 (5.5)	4.0
Undeveloped land	No	285	5.3 (15.8)	2.0	278	5.3 (15.9)	3.0*
	Yes	6	2.0 (2.1)	1.5	13	3.2 (9.6)	0.0
Designated green space	No	280	4.8 (13.9)	2.0	277	5.2 (16.0)	2.0
	Yes	11	16.0 (39.8)	0.0	14	5.5 (8.2)	1.5
**RESIDENTIAL LAND USES PRESENT**							
Residential land use	No	266	4.5 (14.1)	2.0	259	4.5 (14.2)	2.0
	Yes	25	13.3 (26.7)	4.0*	32	11.6 (23.8)	5.0*
Abandoned homes	No	265	5.2 (16.4)	2.0	282	5.3 (15.9)	2.0
	Yes	26	5.5 (4.7)	4.0*	9	3.9 (3.4)	3.0
Multi-unit homes (2–4 units)	No	**236**	**5.0 (17.3)**	**1.0**	270	5.1 (16.2)	2.0
	Yes	**55**	**6.2 (4.7)**	5.0**	21	6.9 (5.1)	6.0**
Single-family homes	No	45	15.4 (37.2)	5.0**	**49**	**14.6 (35.7)**	6.0**
	Yes	246	3.4 (4.6)	2.0	**242**	**3.4 (4.6)**	**2.0**
Apartments (>4 units, 1–4 stories)	No	261	5.2 (16.4)	2.0	260	4.3 (9.7)	1.5
	Yes	30	6.0 (6.0)	5.0	31	13.6 (38.5)	6.0**
**PARKING FACILITIES PRESENT**							
Parking allowed	No	273	5.5 (16.9)	3.0	240	5.2 (16.9)	2.0
	Yes	55	3.8 (7.1)	1.0	51	5.4 (7.8)	3.0
On-street parking	No	73	4.6 (7.5)	1.0	82	5.8 (7.9)	3.0
	Yes	218	5.5 (17.6)	2.0	291	5.0 (17.8)	2.0
Small lot or garage	No	236	3.7 (5.4)	1.0	239	5.1 (17.0)	2.0
	Yes	55	12.1 (33.7)	4.0**	52	5.8 (6.6)	4.0*
Medium/large lot or garage	No	264	4.6 (14.3)	2.0	266	4.8 (14.3)	2.0
	Yes	27	12.0 (25.1)	6.0**	25	9.9 (26.1)	4.0
**PUBLIC RECREATIONAL FACILITIES PRESENT**							
Park with equipment	No	283	5.2 (15.9)	2.0	286	5.2 (15.8)	2.0
	Yes	8	6.4 (4.0)	6.0*	5	7.0 (2.8)	8.0*
Sports/playing field	No	286	5.2 (15.8)	2.0	283	5.2 (15.9)	2.0
	Yes	5	7.0 (6.6)	8.0	8	6.3 (5.6)	7.5
**NON-RESIDENTIAL LAND USES PRESENT**							
Non-residential land use	No	147	7.3 (21.5)	3.0*	119	8.5 (23.5)	4.0**
	Yes	144	3.1 (4.4)	1.5	172	3.0 (4.7)	1.0
Abandoned buildings	No	276	5.2 (16.1)	2.0	276	5.1 (16.0)	2.0
	Yes	15	6.3 (5.6)	6.0*	15	7.1 (9.2)	3.0
Small grocery or convenience store	No	278	5.2 (15.9)	2.0	280	5.2 (15.9)	2.0
	Yes	13	6.6 (9.1)	3.0	11	5.7 (8.5)	3.0
Food establishment	No	272	3.7 (5.0)	2.0	276	4.4 (9.3)	2.0
	Yes	19	27.3 (55.1)	6.0**	15	21.8 (55.5)	4.0
Bank	No	286	4.4 (9.0)	2.0	285	4.3 (9.2)	2.0
	Yes	5	51.4 (94.4)	6.0	6	48.2 (84.8)	17.5*
Church	No	264	5.3 (16.4)	2.0	268	5.4 (16.3)	2.0
	Yes	27	4.4 (5.5)	3.0	23	3.3 (4.0)	3.0
Schools	No	**275**	**5.1 (16.0)**	**2.0**	**275**	**5.1 (16.0)**	**2.0**
	Yes	**16**	**7.8 (7.4)**	5.5*	**16**	**7.6 (7.1)**	6.5**
Strip mall	No	279	5.1 (15.9)	2.0	279	5.1 (15.9)	2.0
	Yes	12	7.8 (11.0)	4.5	12	7.9 (11.0)	3.5
Large office building	No	261	4.7 (14.4)	2.0	260	4.0 (5.6)	2.0
	Yes	30	10.3 (24.0)	3.5	31	16.0 (44.4)	4.0
**PUBLIC TRANSPORTATION AVAILABLE**							
Transit	No	261	3.5 (4.9)	1.5	267	4.3 (9.5)	2.0
	Yes	30	20.2 (44.5)	7.0**	22	16.4 (46.0)	5.0*
**STREET CHARACTERISTICS VISIBLE**							
Posted speed limit	No	210	4.5 (10.5)	2.0	215	4.6 (10.6)	2.0
	Yes	81	7.2 (24.4)	3.0	76	7.0 (25.1)	3.0
Posted special speed zone	No	281	5.2 (15.9)	2.0	280	5.3 (16.0)	2.0
	Yes	10	6.1 (4.2)	6.0*	11	3.9 (3.5)	6.0
Marked lanes	No	**204**	**3.6 (5.4)**	**1.0**	199	3.4 (4.9)	1.0
	Yes	**87**	**9.1 (27.2)**	4.0**	92	9.3 (26.6)	4.0**
Median or island	No	280	5.2 (15.9)	2.0	280	5.1 (15.8)	2.0
	Yes	11	5.6 (6.0)	5.0	11	9.7 (10.7)	6.0**
Turn lane	No	269	4.4 (9.5)	2.0	270	4.4 (9.6)	2.0
	Yes	22	15.3 (46.1)	4.5	21	15.9 (46.9)	5.0*
Crosswalk	No	246	3.5 (4.8)	1.5	254	3.5 (4.9)	2.0
	Yes	45	15.0 (37.1)	5.0**	36	17.4 (41.1)	6.0**
Walk/do not walk signal	No	272	3.8 (5.3)	2.0	267	3.6 (5.1)	2.0
	Yes	19	26.1 (55.3)	6.0**	24	23.3 (49.3)	6.5**
Traffic calming device	No	281	5.3 (15.9)	2.0	284	5.3 (15.9)	2.0
	Yes	10	3.2 (2.5)	2.5	7	4.1 (3.1)	5.0
Cul-de-sac	No	274	5.3 (16.1)	2.0	279	5.3 (16.0)	2.0
	Yes	17	4.2 (6.9)	0.0	12	4.3 (7.1)	0.0
**QUALITY OF THE ENVIRONMENT**							
Commercial building adjacent to sidewalk	No	226	3.5 (4.7)	2.0	217	3.4 (4.8)	1.0
	Yes	65	11.4 (31.4)	4.0*	74	10.7 (29.4)	4.0**
Any amenities	No	157	3.6 (4.5)	2.0	204	3.4 (5.7)	1.0
	Yes	27	23.1 (46.6)	7.0**	5	70.2 (100.9)	0.0
Graffiti or broken windows	No	254	5.1 (16.7)	1.5	280	4.7 (14.0)	2.0
	Yes	37	6.3 (4.9)	6.0**	11	19.5 (37.8)	8.0*
Litter or broken glass
None/a little		237	5.0 (17.1)	1.0	252	5.3 (16.7)	2.0
Some/a lot		54	6.3 (6.2)	5.0*	38	4.8 (4.9)	4.0**
Tree shade
None/a little		179	4.4 (10.9)	2.0	177	6.0 (19.6)	3.0
Some/a lot		112	6.7 (21.2)	3.0	109	4.1 (5.8)	2.0
Slope along walking area
Flat or gentle		271	5.8 (17.2)	3.0*	255	5.6 (16.7)	2.0
Moderate or steep		57	3.0 (5.7)	1.0	33	2.7 (3.3)	1.0
**PLACE TO WALK OR BICYCLE**							
Sidewalk	No	**104**	**1.9 (4.3)**	**0.0**	**109**	**2.2 (4.7)**	**0.0**
	Yes	**187**	**7.1 (19.1)**	4.0**	**181**	**7.0 (19.3)**	4.0**
Buffer between sidewalk and curb	No	**150**	**3.7 (11.6)**	**1.0**	**158**	**4.8 (18.0)**	**1.0**
	Yes	**141**	**6.9 (19.0)**	4.0**	**132**	**5.8 (12.4)**	3.5**
Trees in buffer	No	**205**	**4.7 (18.1)**	**1.0**	**212**	**4.3 (15.7)**	**1.0**
	Yes	**86**	**6.5 (6.8)**	4.5**	**78**	**7.6 (15.6)**	5.0**
Sidewalk continuous within segment	No	**122**	**2.0 (4.8)**	**0.0**	**129**	**2.3 (4.6)**	**0.0**
	Yes	**169**	**7.6 (19.9)**	4.0**	**161**	**7.6 (20.4)**	4.0**
Sidewalk continuous between segments	No	**128**	**1.9 (4.1)**	**0.0**	**138**	**2.5 (4.9)**	**0.0**
	Yes	**163**	**7.9 (20.3)**	4.0**	**152**	**7.7 (20.9)**	4.0**
Sidewalk width at least 5 ft	No	**183**	**2.4 (4.1)**	**1.0**	205	3.9 (5.4)	2.0
	Yes	**108**	**10.1 (24.5)**	6.0**	84	8.6 (27.8)	3.0
Sidewalk width <3 ft	No	235	5.9 (17.3)	3.0*	231	5.1 (17.4)	1.0
	Yes	56	2.6 (3.7)	1.0	57	6.0 (4.8)	5.0**
Missing curb cuts	No	249	5.5 (16.8)	2.0	237	5.7 (17.2)	2.0
	Yes	42	3.6 (5.4)	2.0	53	3.3 (4.7)	2.0
Major misalignments	No	**221**	**5.2 (17.7)**	**1.0**	240	5.5 (17.2)	2.0
	Yes	**70**	**5.5 (6.1)**	4.0**	50	3.9 (3.9)	3.0
Bike sign or markings	No	279	4.9 (15.8)	2.0	286	5.2 (15.8)	2.0
	Yes	12	13.4 (11.0)	11.0**	3	13.0 (12.1)	6.0*
On-street, paved, marked shoulder	No	261	5.4 (16.4)	2.0	267	5.1 (16.2)	2.0
	Yes	30	4.0 (5.8)	1.5	23	6.7 (8.9)	5.0
Shoulder width at least 4 ft	No	279	5.1 (15.9)	2.0	282	5.2 (15.9)	2.0
	Yes	12	8.2 (7.0)	7.0**	8	7.4 (8.6)	5.5
Shoulder continuous between segments	No	265	5.4 (16.3)	2.0	269	5.1 (16.1)	2.0
	Yes	26	3.7 (6.1)	1.0	21	7.0 (9.2)	5.0

*p < 0.05, **p < 0.01.

*^a^Counts of physically activity individuals was not distributed normally; therefore non-parametric statistics were used*.

*^b^Bolded characteristics also vary by population density*.

The number of individuals engaged in any form of physical activity (total count of physically active individuals) was significantly higher on segments with all non-residential *land use*, including commercial or government buildings (field median = 4.0, *p* < 0.05; imagery median = 4.0, *p* < 0.01), schools and schoolyards (field median = 6.0, *p* < 0.01; imagery median = 7.0, *p* < 0.01), and parks with equipment (field median = 7.0, *p* < 0.05; imagery median = 8.0, *p* < 0.05). However, counts of physically active persons were significantly fewer on segments with only single-family homes and significantly higher on segments with multi-unit homes (2–4 units). Significantly more physically active persons were also observed on segments with a *parking lot or garage* (any size) and *public transportation facilities* (e.g., bus stops).

When assessing *street characteristics*, there were significantly more physically active individuals on segments that had marked lanes, crosswalks, or a walk signal at the intersection. While there were more physically active individuals observed on segments with a median/island and a center turn lane, this relationship was significant only when the segment was categorized as having these characteristics using imagery audits.

Several characteristics relating to the quality of the built environment were also significant predictors of the number of physically active persons observed during audits. More physically active persons were observed on segments with commercial buildings adjacent to the segment. Segments with more graffiti and litter also had significantly more physically active individuals. When amenities were assessed by field audits, there were significantly more active individuals on streets with amenities than without; however, this was not found when assessing streets with imagery audits.

When assessing *sidewalk characteristics*, significantly more active individuals were observed on segments with sidewalks, buffers between the street and sidewalk, and continuous sidewalks within and between segments. Similarly, there was significantly more physical activity observed on segments with designated *bike* route signs.

When assessing if population density (person per square mile) varied by built environment characteristics, 25 variables varied significantly. Of those 25 variables, 13 also varied by physical activity behavior, suggesting population density may be a confounder in the relationship between 13 built environment characteristics and physical activity. These 13 characteristics are highlighted in Table 3.

## Discussion

The growing availability of emerging technology (e.g., Google Street View) is allowing researchers to assess the built environment and directly link it with observations of physical activity behavior. Significant relations between several micro-level built environment characteristics and physical activity behavior were observed. Specifically, more active individuals were observed on segments with destinations (e.g., stores, government offices, and schools) than without destinations. The results are consistent with theories suggesting policy changes in zoning and transportation planning that encourages more walkable communities incorporating mixed land use as recommended by the Task Force for Community Preventive Services and the Transportation Research Board-Institute of Medicine (24, 25).

Additionally, street segments with public transportation facilities (e.g., bus stops) and crosswalks, pedestrian signals, and marked lanes were associated with higher counts of physically active individuals, as were segments with continuous sidewalks or a buffer between the street and sidewalk. These data are also consistent with theories promoting Complete Street policies and Smart Growth principles that encourage transportation planners to design neighborhoods that are accessible for all modes of transportation (26).

In addition to identifying built environment characteristics that predict observed behavior, the results also demonstrate the validity of using omnidirectional imagery technology to audit the built environment. Because agreement between field and image-based audits is not 100% on all items, some streets were classified as having a built environment characteristic using field audits, while the same street was classified as not having that characteristic when using image-based audits (or vice-versa). However, 72% of the characteristics assessed had the same results regardless of auditing method, and there was general agreement regarding which environmental characteristics predicted total physical activity. Items not in agreement (e.g., presence of a turn lane and any amenities) typically demonstrated lower reliability in previous studies (17, 18).

While the results of this study are consistent with the current theory regarding the ways the built environment can be improved to better support physical activity, there are several limitations. First, we cannot determine causality. Because this was an observational study, we do not know if the built environment characteristics encouraged more people to be active on certain segments or if more active individuals were observed on certain segments for other reasons (reverse causality).

Because of this study design, the generalizability of these results is limited. Behavior was only observed in a specific context, and we do not know anything about the other behaviors in which observed persons might be engaged or where they engage in these other behaviors. We do not know if the same people would behave the same way in a different location. Similarly, we did not measure individual factors or personal correlates that may influence behavior.

Our methods did not allow us to test if activity levels on a segment impact built environment characteristics (e.g., does more activity increase litter?) or if increased pedestrian traffic increases the likelihood of city planners and transportation departments to design streets with these characteristics. However, the sampling method employed ensured that we had a similar number of streets based on racial and poverty composition as well as commercial land use. This stratification allowed us to equally distribute these characteristics across our sample. The results suggest that several of the street characteristics are associated with more observed behavior. However, population density may be a confounder in the relationship between 13 built environment characteristics and physical activity. For example, observed behavior could be higher on some streets because there are more people who live or work in that area, not just because of the features of the streets. Future research should sample by population density as well as commercial land use to better assess how density mediates the relationship between physical activity and the built environment.

While we were able to assess a large number of segments, 44 segments (15%) were not audited due to safety concerns of the auditors, problems with identifying the specific segment in the field, or scheduling issues and another 103 segments initially sampled did not have imagery available at the time of data collection. These 147 segments were fairly evenly distributed across six of the eight strata, with very few excluded from majority white, low poverty areas (Table 1). It is unknown how these segments, if included, would have affected the results. However, given the distribution, it is unlikely these streets would have changed the direction of the results.

Additionally, the acquisition date of Street View imagery was not available when the imagery audits for this study were conducted. However, in 2012, Google began providing a stamp indicating the month and year Street View images were acquired. Image dates can change along the same street, and the frequency of image updates is unknown, but researchers implementing these methods in the future are now able to assess the temporal match between observed built environment conditions and physical activity behaviors.

Despite these limitations, there are several strengths to this study. Observational methods for physical activity assessment have several advantages over survey methods. First, they allow researchers to identify the type of activity (e.g., running, walking, and cycling), as well as when and where the activity occurred (13). Second, it removes respondent burden, reduces same-source bias, and places the responsibility for unobtrusive monitoring of physical activity behavior on the researchers. The Block Walk Method used in this study provides data on the number of individuals engaged in different types of physical activity on specific segments, allowing more precise linkage of context and behavior (9, 11, 12). Recent studies have demonstrated improved estimation of the effects of built environment on physical activity when contextual specificity is incorporated (14).

Future research should continue to assess physical activity as it occurs and identify the geographic location of the activity as well as other individual motivators as being active (as exemplified by the recent development of ecological momentary assessments) (27). Additionally, studies that dynamically monitor the intensity and geographic context of physical activity behavior using GPS and accelerometers (14, 28) can integrate the auditing methods used in this study to more closely link behavior to micro-level built environment characteristics that may have a significant influence.

## Author Contributions

All authors have contributed substantially to the design of this study, data collection and analysis, and manuscript preparation. All authors have given final approval of the manuscript to be published and agreed to be accountable for all aspects of the work.

## Conflict of Interest Statement

The authors declare that the research was conducted in the absence of any commercial or financial relationships that could be construed as a potential conflict of interest.

## Footnotes

^1^http://maps.google.com/help/maps/streetview/index.html

^2^http://www.mircrosoft.com/virtualearth
